# Coronaviruses and the human airway: a universal system for virus-host interaction studies

**DOI:** 10.1186/s12985-016-0479-5

**Published:** 2016-02-06

**Authors:** Hulda R. Jonsdottir, Ronald Dijkman

**Affiliations:** Federal Department of Home Affairs, Institute of Virology and Immunology, Länggassstrasse 122, 3012 Bern, Switzerland; Department of Infectious diseases and Pathobiology, Vetsuisse Faculty, University of Bern, Bern, Switzerland

**Keywords:** Human coronavirus, Airway epithelium, ALI, Antiviral compound, Cell tropism

## Abstract

Human coronaviruses (HCoVs) are large RNA viruses that infect the human respiratory tract. The emergence of both Severe Acute Respiratory Syndrome and Middle East Respiratory syndrome CoVs as well as the yearly circulation of four common CoVs highlights the importance of elucidating the different mechanisms employed by these viruses to evade the host immune response, determine their tropism and identify antiviral compounds. Various animal models have been established to investigate HCoV infection, including mice and non-human primates. To establish a link between the research conducted in animal models and humans, an organotypic human airway culture system, that recapitulates the human airway epithelium, has been developed. Currently, different cell culture systems are available to recapitulate the human airways, including the Air-Liquid Interface (ALI) human airway epithelium (HAE) model. Tracheobronchial HAE cultures recapitulate the primary entry point of human respiratory viruses while the alveolar model allows for elucidation of mechanisms involved in viral infection and pathogenesis in the alveoli. These organotypic human airway cultures represent a universal platform to study respiratory virus-host interaction by offering more detailed insights compared to cell lines. Additionally, the epidemic potential of this virus family highlights the need for both vaccines and antivirals. No commercial vaccine is available but various effective antivirals have been identified, some with potential for human treatment. These morphological airway cultures are also well suited for the identification of antivirals, evaluation of compound toxicity and viral inhibition.

## Background

Respiratory diseases caused by human coronavirus infection are of both medical and socio-economic importance. Currently, they are studied in various model systems, ranging from cell lines to animal models. Originally, the importance of HCoVs in the burden of human disease was underestimated and as a result, no general therapy exists to treat coronavirus induced disease in humans. Furthermore, no commercial vaccine is available leaving the human population vulnerable to emerging coronavirus infections. Both the Severe Acute Respiratory Syndrome and Middle East Respiratory Syndrome coronaviruses have recently crossed the species barrier and entered the human population to cause severe disease. In this review, we summarize the current knowledge on human coronavirus infection emphasizing the usefulness of organotypic human airway cultures as a model system.

### Coronaviruses

Coronaviruses (CoVs), a subfamily of the *Coronaviridae* family, are positive strand RNA viruses with the largest genome of all known RNA viruses (≥27 Kb). The genomic RNA is capped, polyadenylated and associated with nucleocapsid proteins within an enveloped virion. The envelope is covered by the characteristic surface glycoprotein that gives the virus particles their characteristic crown-like (latin: corona) appearance [1].

All CoVs share a common genome organization where the replicase gene encompasses the 5′-two thirds of the genome and is comprised of two overlapping open reading frames (ORFs), ORF1a and ORF1b that encode for up to 16 non-structural proteins. The structural gene region, which covers the 3′-third of the genome, encodes the canonical set of structural protein genes in the order 5′ - spike (S) - envelope (E) - membrane (M) and nucleocapsid (N) – 3′. The structural gene region also harbors several ORFs that are interspersed along the structural protein coding genes. The number and location of these accessory ORFs vary between the CoV species [2, 3].

In animals, CoV infections are mainly associated with respiratory and enteric disease and can have large economical impact on the veterinary industry, e.g. Porcine Epidemic Diarrhea Virus (PEDV) causes gastrointestinal disease in pigs [4], Infectious Bronchitis Virus (IBV) causes severe kidney and respiratory disease in chicken [5] and Bovine Coronavirus (BCoV) causes both respiratory disease and diarrhea in cattle [6]. Additionally, CoV infections can have other disease manifestations, such as central nervous system (CNS) involvement, hepatitis and peritonitis [7–10].

In humans, CoV infections are mainly associated with respiratory diseases that are considered to have a large impact on the economy due to reduced productivity of the working population. Currently, 6 coronaviruses that cause disease in humans have been discovered. Four of those are commonly circulating and two have caused epidemics of severe acute respiratory disease.

### Human coronaviruses

The first human coronavirus (HCoV), B814, was described in 1965. In the following years, over 30 additional strains were characterized. Ten of those strains could only be isolated from primary embryonic tracheal organ culture. Others were readily isolated from monolayer cultures and were antigenically related to the prototype strain HCoV-229E. HCoV-OC43, for organ culture 43, was isolated and found to be distinct from the 229E prototype strain [11, 12]. In the subsequent decades, research on HCoVs would center on these two distinct viruses.

However, in 2002, an unknown respiratory illness, termed Severe Acute Respiratory Syndrome (SARS), surfaced in Asia. Research determined it to be caused by a novel coronavirus [13, 14]. At the end of the epidemic, this virus had infected over 8000 people, most in China, and caused 774 deaths [15].

Following the discovery of this virus, two additional CoVs causing human disease were identified. HCoV-NL63 was isolated in the Netherlands in 2004 from an infant with bronchiolitis [16] and HCoV-HKU1 in 2005 from a patient with pneumonia in Hong Kong [17]. In 2012, another respiratory HCoV, Middle East Respiratory (MERS)–CoV, was isolated from a patient with pneumonia in Saudi-Arabia [18]. Unlike SARS-CoV, this virus is still intermittently present in the human population and most recently caused a large outbreak in South-Korea [19]. To date, there have been over 1600 cases and almost 600 deaths related to MERS-CoV infection [20].

### Commonly circulating coronaviruses

Out of the 6 known human coronaviruses, HCoV-229E, HCoV-OC43, HCoV-NL63 and HCoV-HKU1 are commonly circulating in the human population and usually cause general respiratory illness and cold symptoms in healthy individuals [21–23]. Like influenza, these viruses are capable of causing more severe disease in the immunocompromised and the elderly [24]. They infect the human airway from the luminal side and progeny viruses are released from the same side facilitating spread through coughing and sneezing [25, 26]. These coronaviruses are responsible for approximately 5–10% of all upper and lower respiratory tract infections [27–29] but the interactions between them and their natural host cells are poorly understood. Currently, it is hypothesized that most of the human coronaviruses may have originated from bats [30, 31]. For example, HCoV-229E is believed to originate from African hipposiderid bats possibly using camelids as intermediate hosts [32].

### Emerging coronaviruses

In the last 15 years, two coronaviruses have crossed the species barrier and caused severe and fatal disease in humans. SARS-CoV surfaced in 2002 and MERS-CoV in 2012 [13, 14, 18]. As opposed to the commonly circulating viruses, which generally only cause mild respiratory symptoms, these viruses presented with higher case fatality ratios, around 10 and 20–50% respectively [33, 34].

Currently, there is abundant phylogenetic evidence for the bat origin of SARS-CoV, based on sequences of SARS-like viruses found among bats in the recent years [35–37]. The initial transmissions of SARS-CoV from animals to humans were traced back to the live animal wet markets and it was hypothesized that the virus made its way into the human population using the civet cat as an intermediate host. However, successful isolation of SARS-like viruses from bats [38] and the fact that a contemporary bat SARS-like virus can infect human airway cultures [39] suggest that an intermediate host between humans and bat might not have been needed for the transmission of SARS-CoV.

The evolutionary origin of MERS-CoV is less clear but it has been speculated to be bats as well. Characterization of an African bat virus closely related to MERS-CoV shows that both the human and camel strains belong to the same viral species and phylogenetic analysis suggests that MERS-CoV infection in camels predates that in humans, suggesting that camels infect humans and not the other way around. Furthermore, the bat virus roots the phylogenetic tree providing further evidence for the bat origin of MERS-CoV [40]. Additionally, human-to-human transmission, although not robust, seems to happen simultaneously as camel-to-human transmission. Therefore, any further adaptation of MERS-CoV to the human host must be monitored carefully and intermediate hosts identified [41].

Many bat coronaviruses have been identified in the recent years further highlighting the zoonotic potential of this family of viruses [30]. Given the documented history of coronaviruses overcoming the species barrier and causing severe disease in humans, it is important to investigate the zoonotic potential of close evolutionary relatives of common HCoVs in a culture model that recapitulates the aspects of the human airway, e.g. morphology and receptor distribution. It’s important to study the mechanisms of pathogenesis and the evolution of zoonotic viruses in detail in order to identify molecular determinants that affect either transmission or pathogenesis. It’s also important to elucidate whether coronaviruses currently circulating in animals are a potential danger to the human population.

### Human coronavirus receptors and cell tropism

All of the known cellular receptors of HCoVs belong to the same protein family, the membrane ectopeptidases. Interestingly, the catalytic activity of these peptidases is not required for viral entry but rather the co-expression of other host peptidases activates the HCoV spike proteins [42, 43]. It has been established that the human transmembrane serine proteases TMPRSSII and HAT cleave and activate the HCoV-229E, SARS- and MERS-CoV spike proteins during viral entry [44, 45].

Out of the four commonly circulating coronaviruses, HCoV-229E is the only one that infects non-ciliated cells using the human Aminopeptidase N (hAPN) as its receptor [46]. This peptidase is predominantly expressed on non-ciliated cells in the human bronchus [47]. SARS-CoV and HCoV-NL63 both utilize the Angiotensin Converting Enzyme 2 (ACE2) for cellular binding [48, 49]. ACE2 is expressed on ciliated bronchial cells along with endothelial cells and both type I and II alveolar cells [50]. MERS-CoV was found to use a different receptor than SARS-CoV, namely the dipeptyl-peptidase 4 (DPP4) [51]. DPP4 is widely expressed in endothelial cells and various epithelial tissues in the human body [52]. In ex vivo human lung organ cultures, different tropism of SARS- and MERS-CoVs was observed. MERS-CoV can actively replicate in both bronchial and alveolar tissue while SARS-CoV primarily replicates in alveolar tissue [53]. The wide cellular tropism of MERS-CoV might contribute to its associated disease severity and high mortality rate whereas the alveolar replication of SARS-CoV would explain why it generally presents with pneumonia.

The cellular surface receptors for HCoV-OC43 and HCoV-HKU1 are currently unknown but receptor determinants for these two viruses have been identified as N-acetyl-9-O-acetylneuraminic acid and O-Acetylated Sialic acid, respectively [54, 55].

All of these viruses can be successfully cultured and investigated in HAE cultures [56, 57]. The discovery of HCoVs, their receptor usage, cell tropism and receptor binding domain (RBD) is summarized in Table 1.

**Table 1:** Human coronavirus overview

Name	Discovery	Protein Receptor	Tropism	Receptor Binding Domain (RBD)	References
HCoV-229E	1966	Aminopeptidase N (hAPN)	Non-ciliated cells	S407-547	[46, 56, 57, 103, 104]
HCoV-OC43	1967	Unknown^a^	Ciliated cells	Unknown	[56, 105]
SARS-CoV	2003	Angiotensin Converting Enzyme 2 (ACE2)	Ciliated cells	S303-537	[13, 14, 48, 57, 106]
HCoV-NL63	2004	Angiotensin Converting Enzyme 2 (ACE2)	Ciliated cells	S476 -616	[16, 49, 56, 107]
HCoV-HKU1	2005	Unknown^b^	Ciliated cells	Unknown	[17, 56]
MERS-CoV	2012	Dipeptyl-peptidase 4 (DPP4)	Non-ciliated cells	S358-588	[18, 34, 51, 108]

^a^Receptor determinant identified as N-acetyl-9-O-acetylneuraminic acid

^b^Receptor determinant identified as O-Acetylated Sialic acid

Furthermore, established reverse genetic systems for HCoV-229E [58], HCoV-OC43 [59] and HCoV-NL63 [60] allow for controlled virus mutation and fluorescent transgene insertion to better understand the interaction of these viruses with their pulmonary host cells.

### Animal models for human coronaviruses

Traditionally, respiratory viruses are studied in animal models, usually mice and ferrets [48, 61]. However, it is not always possible to correctly recapitulate human infection and disease in animal models. The establishment of transgenic animal models for human disease is attainable when either the virus receptor has been identified, which is not the case for all HCoVs, or when viruses can be adapted to a different host. An adapted human virus may not share the same properties as the original human virus. SARS-CoV was found to replicate naturally in various strains of inbred mice but to enhance clinical signs of disease the hACE2 was introduced into these mice. This resulted in murine models with varying degree of human disease similarity. Since SARS-CoV already replicated in mouse cells, adapting it to the murine host was quite successful. This resulted in three mouse adapted strains that caused disease in mice similar to severe SARS-CoV cases in humans [62].

In an effort to establish a mouse model for HCoV-229E infection transgenic hAPN mice were created. However, the insertion of the hAPN into mouse cells is not enough to establish robust HCoV-229E infection in vivo. Nevertheless, cells isolated from these transgenic animals could be infected in vitro [63, 64].

The emergence of both SARS- and MERS-CoVs emphasized the importance of establishing animal models for human coronaviruses. Currently, a few animal models for MERS-CoV have been established. Mice carry their own variant of the viral receptor DDP4 that differs from the human in regions important for MERS-CoV spike interaction and by replacing this receptor with the human one, MERS-CoV can infect mouse cells but the method of hDPP4 insertion has an effect on the degree of pathogenesis observed in these mice [65, 66]. Various non-human primates (NHPs) can be naturally infected with both SARS- and MERS-CoVs. However, disease presentation and pathogenesis differs between the different subspecies and NHP models are expensive, although ideal to study human infection due to their genetic similarity [62].

To establish a link between the research conducted in animal models and humans, an organotypic airway culture system resembling the human airway epithelium has been developed. This model is a universal platform to study human respiratory viruses [67–70]. They have been used successfully for infection studies with all known human coronaviruses [56, 57]. Furthermore, the cultures can be inoculated with a low infectious dosage to mimic natural infection in the human airway. Whereas, animal models often require both high dosage and artificial inoculation routes.

### Human airway epithelial cell cultures

Organotypic cell cultures are becoming increasingly common. Different cell culture models exist to depict different epithelial tissues [71]. These cultures closely resemble their tissue of origin and contain various different cell types with distinctive roles in the polarized tissue. Currently, various organotypic cell culture models exist to represent the different areas of the human airways. The human lungs span a long anatomical distance and carry out different functions depending on anatomical location [72, 73]. The structure of the epithelium also differs the further you descend into the airways. Tracheal and bronchial epithelium is columnar and pseudostratified, with every cell in contact with the basement membrane, while the epithelium in the alveoli is comprised of a single cell layer to facilitate air-exchange [74].

**Fig. 1:**
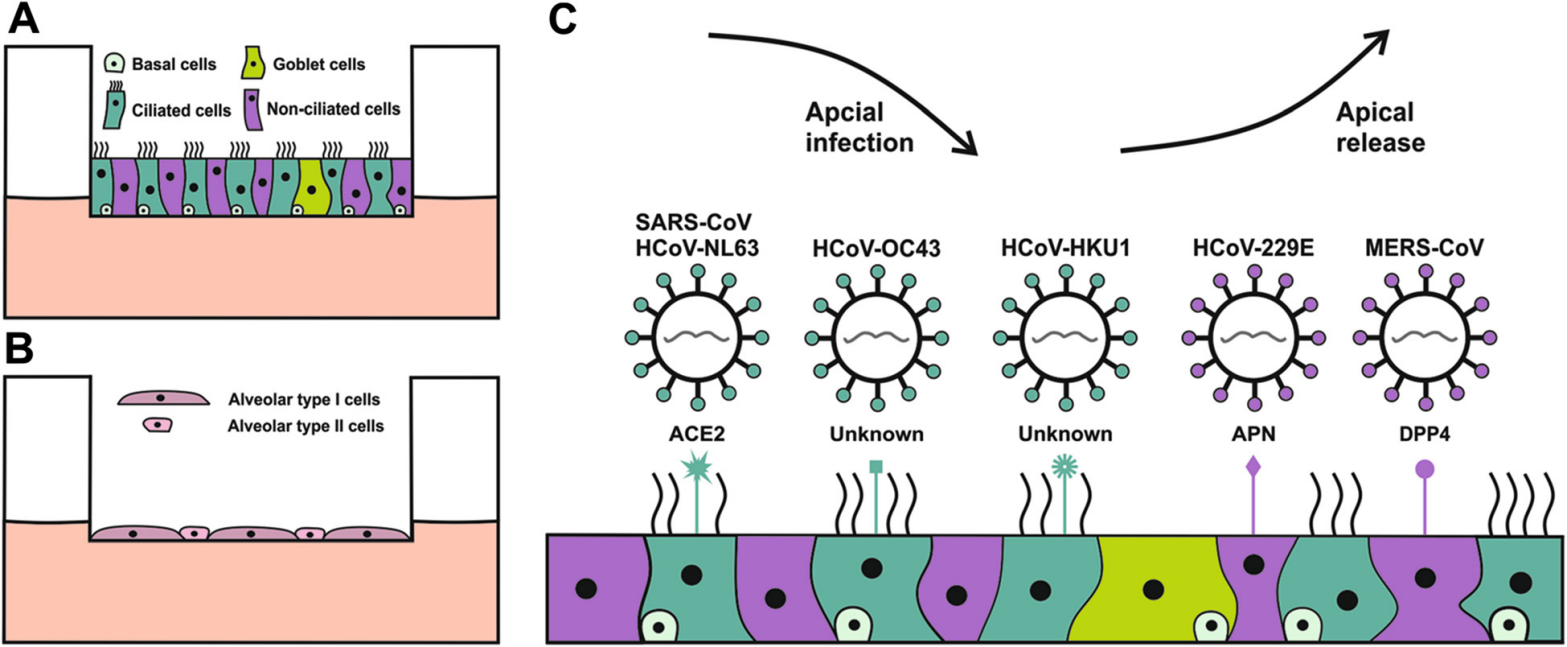
Human airway epithelial cell culture models and HCoV receptor distribution. **a**: Schematic representation of human tracheobronchial cells at air-liquid interface (ALI). They form a pseudostratified epithelial layer containing different cell types. **b**: Schematic representation of human alveolar cells at ALI that form single squamous epithelium containing only two cells types, alveolar type I and II cells. **c**: Illustration of the mode of infection, release and associated cell tropism of the six human coronaviruses (HCoVs) in the human airway epithelial cell culture model. SARS-CoV, HCoV-NL63, HCoV-OC43 and HCoV-HKU1 infect ciliated cells but the receptors for HCoV-HKU1 and HCoV-OC43 are currently unknown. HCoV-229E and MERS-CoV infect non-ciliated cells using different receptors

Tracheobronchial cells are one of the first targets of human respiratory viruses and can be cultured in air-liquid interface (ALI) where the apical side of the cell layer is exposed to air while the basolateral side is submerged in medium. Tracheobronchial cells cultured in that way form a pseudostratified epithelial layer that both morphologically and functionally resembles the human upper conducting airway (Fig. 1a) [75, 76]. After differentiation, these cultures contain many different cell types such as basal, ciliated and goblet cells. They also produce protective mucus, much like in vivo epithelium. When compared to primary bronchial cells in submerged two-dimensional culture, the gene expression of primary ALI cultures differs significantly. However, the expression pattern of primary human bronchial ALI cultures is comparable to that of in vivo epithelium. The human bronchial cell line Calu-3 has been used as a culture model for respiratory epithelium but its gene expression in ALI cultures is more similar to submerged bronchial cell cultures than differentiated epithelium [77]. Additionally, Calu-3 cells respond differently to MERS-CoV infection compared to primary HAE cultures. During infection in Calu-3 cells, profound apoptosis was detected within 24 h of infection [78] while infection of primary HAE cultures does not result in any disruption of the cell layer [57]. Therefore, the primary tracheobronchial ALI culture model is especially fitting for human respiratory virus research since it accurately recapitulates the primary entry point for these viruses. By using these cultures, virus replication and host interactions can be studied in natural target cells. Further establishing the usefulness of this system HCoV-HKU1 was propagated for the first time in ciliated cells of bronchial HAE cultures in 2010 after culturing it in conventional cell lines had failed [26].

Alveolar epithelial ALI cultures (Fig. 1b) can also be used for virus-host interaction studies and are especially applicable when a viral infection causes pneumonia and alveolar damage [79]. HCoV-HKU1 has also been propagated in alveolar HAE cultures and exhibits a strong tropism for alveolar type II cells and causes large syncytia formation upon infection [80].

When compared to traditional two dimensional cell cultures, the HAE cultures are more cumbersome and their preparation is time consuming but they do have an advantage over traditional monolayer cell cultures when it comes to virus-host interaction studies. Different types of ALI cultures used for virus research are summarized in Table 2.

**Table 2:** Different types of ALI cultures used in coronavirus studies

Cell types	Origin	HCoVs	Features	References
Primary bronchial	Trachea, Bronchus	All HCoVs	Differentiated pseudostratified epithelium, many cell types	[56, 57]
Calu-3	Sub-mucosal glands (adenocarcinoma)	MERS-CoV, SARS-CoV	Single or polarized epithelium, one cell type	[109, 110]
Primary alveolar	Alveoli	MERS-CoV, SARS-CoV, HCoV-HKU1	Differentiated squamous epithelium, two cell types	[79, 80, 111]

### Innate immunity

Within the respiratory epithelium the innate immune system has a major protective role as the first line of defense against respiratory pathogens. In particular, the interferon (IFN) system orchestrates hundreds of different cellular effector proteins that (i) protect the epithelial barrier by altering the physiological and cellular environment, (ii) impair virus propagation, spread and transmission, and (iii) shape the host’s adaptive immune response. Recent publications have demonstrated that the innate immune system is functional in the HAE cell culture system and that most pathogen recognition receptors are expressed and up-regulated upon treatment with exogenous stimuli [57, 81].

In general, HCoVs do not elicit a strong innate immune response in primary target cells of the human airway early during infection. Despite the presence of all major pathogen recognition receptors, no elevated expression of IFN beta, pro-inflammatory cytokines or interferon stimulated genes can be observed up to 12 h post-infection in HAEs infected with HCoV-229E, MERS- or SARS-CoVs [57]. This is most likely due to the intrinsic CoV properties harbored in the replicative non-structural proteins that actively aid in avoiding recognition by the host innate immune system. For example, the 5′ termini of the viral mRNA are capped making them indistinguishable from the host cellular mRNAs and no longer detectable by cellular sensors. Furthermore, CoV replication is associated with the appearance of double membrane vesicles (DMVs) in the host cell cytoplasm, which might serve as a protective shield for viral RNA to prevent recognition by cytoplasmic RNA sensors [82–85].

In addition to the non-structural proteins, various CoV accessory proteins have been discovered to inhibit interferon signaling at different stages of the host innate immune response. For example, MERS-CoV accessory protein 4a inhibits innate antiviral signaling by suppressing the activation of MDA5 and RIGI [86, 87] whereas 4b inhibits the induction of the IFN-beta promoter [88]. While ORF 4a and 4b are IFN antagonists in the genome of MERS-CoV, SARS-CoV ORF3b antagonizes IFN signaling through MAVS/RIGI [89]. Whereas SARS-CoV ORF6 disrupts IFN signaling by blocking the nuclear translocation of STAT1 [89, 90]. These discoveries highlight that HCoVs employ similar yet different strategies to evade the innate immune response during infection in the respiratory epithelium.

### Therapy

Despite that respiratory infections with HCoVs can result in severe respiratory disease there are currently no effective prophylactic or therapeutic treatment options available. However, the emergence of novel coronaviruses has emphasized the need to develop effective treatment options. For example, vaccines using the spike proteins of both SARS- and MERS-CoVs have proven protective in animal models [91, 92] suggesting that a vaccine against HCoVs for human use might be achievable.

Additionally, various drugs that inhibit HCoV infection at different stages of the replication cycle have been reported and some could potentially serve as treatment options for HCoV associated severe respiratory disease. For example, patients presenting with severe respiratory disease, caused by SARS- or MERS-CoVs, are generally treated with steroids and interferon, sometimes in combination with the antiviral drug Ribavirin [93–96]. This treatment, however, is not especially effective highlighting the need for HCoV specific antivirals. Many different compounds have been determined to have anti-HCoV activity. For example, protease inhibitors which suppress HCoV entry [97–99], Cyclosporin A (CsA) treatment blocks the replication of coronaviruses from all subgroups [100] and non-immunosuppressive derivatives of CsA represent a possible therapeutic option for both human and animal CoV infections.

HCoV infection can also be inhibited by pre-treating HAE cultures with either recombinant IFN alpha or lambda [57]. Similar effect has also been shown for recombinant IFN alpha and beta which could inhibit MERS-CoV in ex vivo lung cultures [53]. As previously described, IFN treatment of active HCoV infection is not particularly effective in vivo. Therefore, the use of IFN in humans might be limited to prophylactic treatment of exposed persons and/or health care workers treating infected patients.

Screenings of compound libraries have also resulted in the identification of some HCoV specific antivirals. For example, a novel small compound inhibitor (K22) has been identified, and showed to be effective against a broad spectrum of CoVs and could inhibit both HCoV-229E and MERS-CoV in HAE cultures [101]. Additionally, HCoV-NL63 has been inhibited in HAE cultures with polymer-based compounds [102].

To date, most treatment and inhibitor studies have been conducted in HCoV susceptible cell lines. However, the HAE cultures represent an ideal system to test the application and efficacy of those already identified, and new, antiviral compounds against HCoVs in cells that represent the primary site of replication. Furthermore, the HAE cultures are heterogenous, containing many different cellular sub-populations, and would allow for the evaluation of compound toxicity and effect in a differentiated layer similar to human airway epithelium. Compounds already shown to inhibit HCoVs in cell lines should be applied to HAE cultures as well before any animal or human trials.

## Conclusions

HCoV induced respiratory diseases are of both medical and socio-economic importance. The emergence of SARS- and MERS-CoV and the yearly circulation of the four common HCoVs highlight the importance of elucidating the different mechanisms employed by HCoVs to evade the host immune system as well as identifying antiviral compounds and human vaccine candidates. The HAE culture system is based on primary human cells offering a unique platform to study respiratory viruses in cells representing the primary entry point of these viruses, bronchial epithelial cells, or investigate the interaction of HCoVs and the distal airways, in type I and II alveolar cells. Additionally, the inclusion of airway epithelial cultures for other species enables the study of zoonosis and animal-to-human transmission. Currently, many aspects of HCoV infection and pathogenesis remain to be determined. The HAE culture system, both tracheobronchial and alveolar, represents a unique platform to study virus-host interaction in natural target cells at the molecular level. These cultures are becoming more common and more relevant to HCoV research. Especially, for those viruses for which there is no animal model, as they provide an organotypic substitute for virus – host interaction studies.

## Abbreviations

ALI: Air-Liquid Interface
BCoV: Bovine Coronavirus
CNS: Central Nervous System
CoV: Coronavirus
CsA: Cyclosporin A
DMV: Double Membrane Vesicles
FDA: Food and Drug Administration
hACE2: human Angiotensin Converting Enzyme 2
HAE: Human Airway Epithelium
hAPN: human Aminopeptidase N
HAT: Human Airway Trypsin-like protease
HCoV: Human Coronavirus
hDPP4: human Dipeptyl Peptidase 4
IBV: Infectious Bronchitis Virus
IFN: Interferon
MDA5: Melanoma Differentiation-Associated protein 5
MERS: Middle East Respiratory Syndrome
NHP: Non-Human Primate
ORF: Open Reading Frame
PEDV: Porcine Epidemic Diarrhea Virus
RBD: Receptor Binding Domain
RNA: Ribonucleic Acid
SARS: Severe Acute Respiratory Syndrome
TMPRSSII: Transmembrane Protease, Serine 2
